# From Production to Application: Postbiotics in Meat, Meat Products, Other Food Matrices, and Bioactive Packaging

**DOI:** 10.3390/foods15030501

**Published:** 2026-02-01

**Authors:** Miłosz Trymers, Patryk Wiśniewski, Katarzyna Tkacz, Arkadiusz Zakrzewski

**Affiliations:** Department of Food Microbiology, Meat Technology and Chemistry, Faculty of Food Science, University of Warmia and Mazury, Plac Cieszyński 1, 10-726 Olsztyn, Poland; milosz.trymers@uwm.edu.pl (M.T.); ktkacz@uwm.edu.pl (K.T.); arkadiusz.zakrzewski@uwm.edu.pl (A.Z.)

**Keywords:** lactic acid bacteria, biopreservation, food safety, cell-free supernatants, clean label

## Abstract

Postbiotics represent a promising strategy for reconciling increasing consumer demand for clean-label foods with the need to maintain high microbiological safety standards. The present review analyzed the applications of postbiotics in meat products, other food matrices and bioactive packaging, with particular emphasis on their production methods, compositional analysis and antimicrobial properties. Available evidence indicates that postbiotics offer important technological advantages over live probiotics, including enhanced stability during processing and storage and the absence of viable cells, which facilitates their integration into established food quality and safety control systems. The reviewed studies show that postbiotics produced mainly via fermentation with selected lactic acid bacteria and subsequently stabilized, most often by freeze-drying, exhibit pronounced antimicrobial activity in diverse food matrices, particularly meat and dairy products. Their ability to inhibit the growth of major foodborne pathogens, such as *Listeria monocytogenes*, *Staphylococcus aureus*, *Escherichia coli*, and *Salmonella* spp., highlights their potential as effective biopreservatives contributing to shelf-life extension and improved microbiological safety. From an industrial perspective, postbiotics can be implemented within the framework of hurdle technology and incorporated into active packaging systems and edible coatings. The wider use of postbiotics in industry remains limited by regulatory uncertainty and methodological diversity. Key challenges include inconsistent taxonomic/strain reporting, divergent methods of inactivation and final processing (which alter bioactive profiles), lack of standardized composition and potency testing, and limited food matrix validation and toxicological data. To eliminate these gaps, regulatory definitions and labelling should be harmonized, and guidelines for production and reporting (strain identity, inactivation parameters, preservation method), and targeted safety and shelf-life testing are recommended. These steps are necessary to translate the documented antibacterial and antioxidant properties of postbiotics into industrial applications.

## 1. Introduction

Consumers are becoming increasingly aware of food processing methods and the composition of food products. As a result, they tend to choose items that align with the clean-label concept, which is associated with short, transparent, and easily understandable ingredient lists [1]. This trend has contributed to increased consumer interest in the use of natural preservatives, herbs, spices, and microbial metabolites [2]. Among these alternatives, postbiotics represent an innovative strategy for improving food quality and safety [2].

Meat processing involves the use of, among others, nitrate (E251) and sodium nitrite (E250) as well as synthetic antioxidants, including butylated hydroxytoluene (BHT, E321), butylated hydroxyanisole (BHA, E320) and tert-butylhydroquinone (TBHQ, E319), but their use is regulated due to potential health risks [3,4]. Nitrites can react with amines, forming nitrosamines with documented carcinogenic properties [5]. Moreover, BHT, BHA, and TBHQ are associated with the risk of allergic reactions and toxicological effects with long-term consumption [6,7]. In response to these consumer challenges related to the clean label trend, postbiotics may represent an innovative strategy for improving the quality and safety of food products [2].

Postbiotics, according to the consensus definition of the International Scientific Association of Probiotics and Prebiotics (ISAPP), are “a preparation of inanimate microorganisms and/or their components that confers a health benefit on the host” [8]. Unlike probiotics, postbiotic preparations do not contain viable microbial cells in the final product [9]. Compounds typically classified within this group include bacteriocins and bacteriocin-like compounds (BLC), organic acids, fatty acids, hydrogen peroxide (H_2_O_2_), ethanol, and reuterin, many of which have documented antimicrobial and antioxidant properties [10]. Numerous studies have demonstrated that postbiotics exhibit a broad spectrum of biological activity, including antioxidant effects [11] and antimicrobial properties against key food-borne pathogens, such as *L. monocytogenes* [11,12], *S. aureus* [11,13], *E. coli* [11], *Salmonella* Typhimurium [13], and *Clostridium botulinum* [14]. Their ability to inhibit both spoilage microorganisms and foodborne pathogens makes them a promising tool in modern food preservation strategies [10]. In addition to their antimicrobial functions, postbiotics have been shown to reduce lipid oxidation, which is a key aspect in extending the shelf life of food products [11,15,16]. The metabolites they contain can neutralize reactive oxygen species or chelate pro-oxidative metal ions, thereby slowing down the initiation and propagation of lipid peroxidation reactions [10]. As a result, their use not only inhibits the growth of undesirable microbiota but also improves the oxidative stability of food, which can be important for maintaining the sensory quality and nutritional value of products during storage. The activity of postbiotics results from the presence of various bioactive compounds, such as bacteriocins, organic acids, phenolic compounds, and flavonoids [14]. Bacteriocins, which are peptides or proteins synthesized by bacteria and archaea, exhibit strong antimicrobial activity by binding to lipid II (a chief transporter of peptidoglycan subunits from the cytoplasm to the cell wall) and forming pores in the cytoplasmic membrane, leading to destabilization of the membrane potential, inhibition of ATP synthesis, and bacterial cell death [17]. Organic acids (lactic, acetic, propionic) have an antimicrobial effect due to the diffusion of the undissociated form into the cell, lowering the intracellular pH, metabolic disturbances, and ATP depletion as a result of osmotic stress [18]. Phenols, polyphenols, and flavonoids, including catechins and quercetin, exhibit antimicrobial activity by damaging cell membranes and inhibiting the activity of enzymes key to microbial metabolism, while also acting as powerful antioxidants that limit the oxidation of meat lipids [19]. In recent years, there has been growing interest in the use of postbiotics in the food sector, resulting from the increasing demand for food free from synthetic preservatives [14].

Lactic acid bacteria (LAB), many of which hold GRAS (Generally Recognized as Safe) or QPS (Qualified Presumption of Safety) status, are the most commonly used microorganisms in the production of postbiotics. Among the most commonly used species are *Lactiplantibacillus plantarum*, *Lactobacillus acidophilus*, *Pediococcus acidilactici*, *Latilactobacillus sakei*, *Latilactobacillus curvatus*, *Ligilactobacillus salivarius*, and *Lactobacillus delbrueckii* subsp. *bulgaricus* [10,20]. In recent years, there has also been growing interest in microorganisms outside the LAB group. These include yeasts (*Saccharomyces boulardii*) and bacteria such as *Enterococcus faecium*, *Eubacterium hallii*, or *Akkermansia muciniphila* [20].

Postbiotics show greater stability during food processing than live probiotic or protective cultures. Live probiotics are sensitive to pH and temperature, which limits their shelf life in food matrices. In addition, the presence of viable protective cultures increases the total viable count (TVC), which may be misinterpreted as product spoilage [14,21,22]. For these reasons, postbiotics are increasingly investigated as an alternative biopreservation strategy. The application of postbiotics responds to key needs of the food industry, including delaying spoilage processes and ensuring the microbiological safety of products, in line with the trend towards natural and sustainable food preservation methods [14].

Therefore, this review focuses on the production methods, compositional characteristics, and antimicrobial properties of postbiotics, with particular emphasis on their application in meat products and other food matrices. In addition, it addresses the incorporation of postbiotics into edible films and active packaging systems as platforms for delivering bioactive components and extending product shelf life.

## 2. Regulations Concerning Postbiotics

Industry-oriented analyses highlight that postbiotics are currently variably classified across jurisdictions (e.g., as food ingredients, novel ingredients, supplements, or therapeutic agents), which leads to divergent data requirements and approval pathways, complicating commercialization [23]. Such regulatory heterogeneity reflects the absence of a dedicated postbiotic category in most jurisdictions, forcing manufacturers to navigate multiple existing legal frameworks depending on the intended use and product format [23,24].

In the European Union (EU), food regulations cover both general principles for ensuring consumer safety and specific requirements for the use of health claims [24]. However, the lack of a uniform position on the definition of postbiotics is a significant obstacle to the development of a coherent and unambiguous legal framework for this group of ingredients [8,24]. At present, the EU has not established any postbiotic-specific regulation, so these products must be assessed within existing categories such as foods, food supplements, medicinal products, or novel foods, depending on their intended use [23,24].

Products containing inactivated microorganisms are usually assessed on a case-by-case basis, most often under Regulation (EU) 2015/2283 [25] on novel foods and in the context of health claim regulations. In practice, the safety assessment may be facilitated by granting QPS status to the species/strain, while the lack thereof requires a detailed assessment at the strain and inactivation process level [26]. For example, EFSA continues to update the QPS list [27] and makes guidelines on application requirements for novel foods publicly available, as well as issuing individual opinions on the safety of preparations containing non-viable microorganisms (e.g., opinion on inactivated *Saccharomyces cerevisiae* as a feed additive [28]. EFSA has emphasized that, for non-viable microbial preparations, the inactivation process, batch-to-batch reproducibility, and full compositional characterization are key determinants of safety [26,29].

If a product is classified as a novel food, an extended safety assessment is required, including toxicological data [29]. EFSA has assessed the safety of several preparations of non-viable bacteria, for which the characteristics of the inactivation process were a key element of the safety evaluation. These preparations did not obtain QPS status in their live form, but some of them were positively assessed for use as novel foods after obtaining the consent of the European Commission [26,29].

Importantly, this lack of postbiotic-specific legislation is not unique to the European Union. In other major markets, including North America, Australia/New Zealand, and Japan, products based on inactivated microorganisms are likewise regulated within existing legal categories (such as food ingredients, novel foods, supplements, or foods with health claims) rather than under a dedicated postbiotic framework, resulting in jurisdiction-specific data requirements, labelling rules, and authorization pathways [23,24].

Because the ISAPP definition is scientific rather than legal, regulatory authorities in all jurisdictions apply their own statutory definitions and evidentiary standards, which creates uncertainty for manufacturers seeking international commercialization of postbiotic products [8].

Consequently, the frequently stated conclusion that the industrial application of postbiotics is “limited by regulations” should be understood as a global issue arising from the absence of harmonized legal definitions, standardized characterization criteria, and aligned regulatory pathways, rather than as a problem restricted solely to the European Union [8,23,24].

## 3. Postbiotic Production

### 3.1. Microorganisms

LAB are the most commonly used in the production of postbiotics. Among the microorganisms used, the dominant strains were *P. acidilactici* [30,31,32] and *L. acidophilus* [33,34,35]. *L. plantarum* [33,36] and *Bifidobacterium animalis* subsp. *lactis* [34,37] were also frequently used. Several studies also used other microorganisms of the *Pediococcus* spp. [32] and *Lactobacillus* spp. without specifying the strain species [38]. In addition, other LAB have also been used in a few cases, such as *Streptococcus salivarius* subsp. *thermophilus* [39], *L. delbrueckii* subsp. *bulgaricus* [39], *Lacticaseibacillus casei* [36], *L. sakei* [40], *Lactiplantibacillus pentosus* [41], *Lactococcus lactis* [32] and *Pediococcus pentosaceus* [32]. Some of the analyzed studies also reported the use of commercially available mixtures of LAB [42] and *Saccharomyces cerevisiae* var. *boulardii* yeast [43].

### 3.2. Postbiotic Production Steps

The fermentation process has a key role in postbiotic production. It is a crucial element that determines the composition and properties of the metabolites obtained [44]. Depending on the applied strategy, either submerged or solid-state fermentation is employed [44]. This process is carried out using selected strains of microorganisms, cultivated under conditions optimized for each strain [44]. The type of fermentation substrate used has a significant impact on the efficiency of the process, the concentration of metabolites produced, and their functional properties and bioactivity [44,45]. This can be a laboratory culture medium (e.g., De Man, Rogosa, and Sharp (MRS) broth) as well as milk proteins, whey, and, in industrial production, cane straw, wheat bran, or corn straw [45,46,47,48]. After the fermentation process is complete, the selection of subsequent processing stages depends on the nature of the target metabolites, which may be intracellular, extracellular compounds, or a combination of both [45].

The fundamental difference between the processing of these two fractions is the need to apply a cell lysis step in the case of intracellular metabolites, which is not required for extracellular metabolites secreted directly into the fermentation broth [45]. The most commonly used methods for obtaining intracellular fractions of postbiotics include thermal processes such as pasteurization, sterilization, and steam heating, which damage the cellular structures of microorganisms, leading to the loss of cell viability and the release of intracellular metabolites. In the case of extracellular metabolites, this stage is omitted [49,50].

Research indicates significant diversity in the conditions for culturing microorganisms used in the production of postbiotics, including both the type of culture media used and the incubation parameters and methods of postbiotics preservation (Table 1). In many studies, the culture medium consisted of dairy products, such as skimmed milk or whey, which are rich sources of nutrients necessary for the intensive growth of lactic acid bacteria [33,34,37,39]. Considerable variability was observed in incubation parameters across studies. Temperatures were strain-dependent and most commonly fell within 30–40 °C, with 37 °C used most frequently. Aerobic conditions predominated [30,33,34,35,37,38,39,41,42,43], while anaerobic cultivation was reported only sporadically [31,32,36,40]. Media were often enriched with growth-promoting additives, most notably lactose (0.18–3.9%) [35,37] and yeast extract at variable levels [33,34]. These supplements are added to increase the metabolic activity of bacteria and improve the efficiency of bioactive compound synthesis.

The next stage of the process is to separate the cell biomass from the fraction containing metabolites by centrifugation, followed by filtration [51]. The postbiotics obtained can be further subjected to additional preservation and modification processes, such as encapsulation, freeze-drying, or concentration [45]. However, liquid-phase removal and drying methods differ in their impact on bioactive constituents: thermal evaporation and high-temperature drying frequently lead to loss of volatile bioactives, while freeze-drying is generally considered a gentler approach and is commonly used to better preserve labile biomolecules. Nevertheless, losses of particular volatile metabolites may still occur during freezing and subsequent primary/secondary drying depending on compound volatility, product matrix, and process parameters; therefore, the choice of preservation and encapsulation strategy should be made case-by-case to retain functional activity [45,52,53]. In the majority of studies, the obtained postbiotic preparations were subjected to freeze-drying [33,35,36,37,38,39,40,42]. The process was carried out under low-pressure conditions, while temperature and process duration varied depending on the protocol. Such a wide variation in cultivation and preservation parameters indicates a lack of a standardized protocol, which makes it difficult to directly compare the results obtained by different researchers and, at the same time, highlights the need to develop technological standards that enable the repeatable and effective production of postbiotics with specific functional properties.

However, it should be emphasized that it is not only processes such as steaming or freeze-drying that can lead to the loss of volatile bioactive compounds. Meat processing can also compromise their stability through different pathways. The mechanisms and effects of these interactions depend on the nature of the compound, the process parameters, and the meat matrix [54]. Curing (sodium chloride (NaCl)) ± nitrites/nitrates) inhibits the growth of bacteriocin-producing cultures and reduces the production of postbiotics, as demonstrated in a study by Papagianni et al. (2013) [55]. Chemical reactions involving nitrites and changes during maturation or curing can also modify amino and peptide residues, altering the biological activity and detectability of bioactive peptides [56]. Heat treatment (pasteurization, cooking, baking) usually causes protein denaturation and degradation of many thermostable peptides, which can lead to the loss of biological activity of various compounds. At the same time, bacteriocins (e.g., nisin) exhibit significant thermal resistance, which allows them to be used in selected thermal processes or after prior stabilization [57]. Freezing and freeze–thaw cycles also affect muscle tissue structure and protease activation, which in turn can lead to modification and partial degradation of peptides and changes in the antioxidant and antibacterial activity of postbiotics [58]. However, the number of cycles and the freezing rate should be taken into account, as these determine the scale of the changes. Although drying and smoking processes lead to the concentration of compounds, they also cause the oxidation of lipids and proteins and additional Maillard reactions, which may also degrade some bioactive peptides [59].

The use of postbiotics in meat products, therefore, requires the adjustment of strategies (selection of strain/compound, timing of application—before/after processing, use of carriers/molecular protection) and validation of stability and functional activity in the target matrix and under target technological conditions using combined analytical methods.

**Figure 1:**
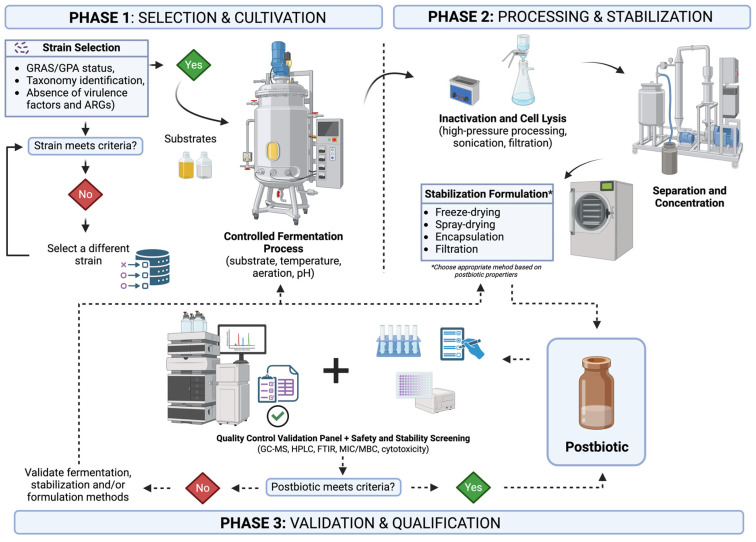
Diagram of the postbiotic production process, from strain selection to the final product. Created in BioRender. Zakrzewski, A. (2026) https://BioRender.com/efz7e6e (accessed on 21 January 2026).

A summary of the postbiotic production process, including key decision points shown in Figure 1.

### 3.3. Standardization Challenges

In the production of postbiotics, there is considerable interchangeability of parameters, including the selection of strains (e.g., *S. thermophilus* [39], *L. delbrueckii* subsp. *bulgaricus* [39], *B. animalis* subsp. *lactis* [37], *L. acidophilus* [33,35], *L. plantarum* [33,36], *Pediococcus* spp. [30,31], *S. boulardii* [43]), the type of medium (skimmed milk [33,35,37,39], whey [34,35,36,37,39], MRS [31,32,36,38,40,41,42], TSB [30], YMB [43]), and incubation and culture conditions (temperatures ~30–46 °C, incubation times ~20–68 h, aerobic or anaerobic conditions), while using various nutritional supplements (including lactose in concentrations of 0.18–3.9% and yeast extract 1.0%), which suggests different metabolic conditions affecting the profile of metabolites produced—Table 1. In addition, the differentiation concerns the final forms and processing procedures (including different lyophilization parameters, as well as the direct use of CFS, preparation of postbiotic solutions, 10%/50%/100% dilutions, rehydration or dissolution of 1 g of material in 1 mL of phosphate buffer) implies significant differences in the stability, storage and methods of application of the final material—Table 1. Therefore, the comparability of results between studies is limited without the standardization of key production parameters, which justifies the need for precise reporting: composition and type of matrix, incubation temperature and time, aerobic/anaerobic conditions, supplements used with indication of concentrations and details of final processing, as well as the obligation to characterize the quality and quantity of the final product in order to link production conditions to the biological effect and enable the standardization of protocols adapted to the specific strain and matrix used.

## 4. Methods for Analyzing the Composition and Effectiveness of Postbiotics

The chemical composition of postbiotic preparations is complex and variable, due to differences in strain metabolism and culture conditions. As a result, various analytical methods are required for their comprehensive characterization [36,60]. Understanding which bioactive components are present in postbiotic preparations is essential to elucidate their mechanisms of action. The identification of individual compounds, such as organic acids, phenols, or flavonoids, allows the chemical structure to be linked to the observed biological activity [14]. Therefore, multiple analytical techniques are typically combined to achieve a thorough chemical characterization [36]. Organic acids (e.g., lactic acid, acetic acid), phenolic compounds, and flavonoids are the most common and dominant low-molecular-weight components of postbiotics [61]. Organic acids form the basis of the CFS fraction and are responsible for a significant part of the antimicrobial activity, while phenols and flavonoids, although in lower concentrations, significantly support antioxidant, antibacterial, and anti-biofilm activity [61,62]. Other classes of compounds present in postbiotics (bacteriocins, hydrogen peroxide, biosurfactants) also participate in the activity, but these three groups are the most popular [63].

### 4.1. Postbiotic Composition Analysis

#### 4.1.1. Spectrophotometric Methods

Spectrophotometric methods are commonly used for the quantitative determination of the main groups of bioactive compounds in postbiotics. The Folin–Ciocalteu reagent method is used to determine the total phenolic content (TPC) [14]. These methods allow for a quick assessment of the antioxidant potential of postbiotic preparations.

#### 4.1.2. Chromatographic Methods

Gas chromatography with mass spectrometry (GC-MS) allows for a much broader chemical profiling of postbiotics [36,60]. This method reveals a wide range of bioactive metabolites whose presence and concentration are highly variable, and whose chemical signatures depend fundamentally on the specific microbial strain, culture conditions, and analytical methods used for their identification [60,64]. This variability in metabolic profiles is directly linked to the functional properties of postbiotics, such as their antimicrobial activity.

A detailed analysis reveals fundamental metabolic differences between various microorganisms, which directly affect their potential applications. The metabolomic profile of the postbiotic from *S. cerevisiae* var. *boulardii* ATCC MYA-796 includes unique compounds such as Ergotaman-3′,6′, 18-trione, and 5,10-diethoxy-2,3,7,8-tetrahydro-1H,6H-dipyrrolo [1,2-a:1′,2′-d]pyrazine, which have documented antibacterial, anti-inflammatory, and antioxidant properties [43]. This profile differs significantly from the metabolites generated by probiotic bacteria; for example, 3-phenylmalic acid (PLA), considered a chemical marker for certain strains of *L. plantarum*, was not detected in the yeast-derived postbiotic preparation [43]. Further evidence of this diversity is provided by the profile of *Lactobacillus* spp. RM1, in which 6-octadecenoic acid methyl ester and hexadecanoic acid methyl ester were identified as the main components, differing from the compounds produced by *L. plantarum* K35 [65].

Significant differences in the chemical composition of postbiotics are observed even within the *Lactobacillus* genus, although certain metabolic components remain common. Benzoic acid and cyclopentane are commonly detected in CFS from various strains [60]. Interestingly, pyrrolo [1,2-a]pyrazine-1,4-dione, considered to be a compound with antimicrobial and antioxidant properties [66], has been identified both in supernatants of various *Lactobacillus* spp. strains [60] and in the *S. cerevisiae*–derived postbiotic [43], which may suggest the existence of common metabolic pathways or conserved functional roles. In addition to these common components, there are dominant compounds specific to a given strain. For example, in the postbiotics analyzed in the study by Moradi et al. 2019 [60], compounds such as 6-octadecanoic acid methyl ester (44.13%) predominated. In turn, the analysis of *L. plantarum* and *L. casei* metabolites showed significant production of succinic acid, a key intermediate compound in the Krebs cycle [36]. Some strains, such as *Lactobacillus rhamnosus* GG, exhibit a particularly rich and diverse volatile profile with high levels of acetone and isovaleric aldehyde, especially after processing techniques such as pascalisation (high-pressure processing, HPP) [64].

The identified metabolites are considered to have significant functional importance, primarily antimicrobial activity. For example, long-chain fatty acids (C16-C18) exhibit antifungal properties against pathogens such as *Rhizoctonia solani*, as confirmed by Walters et al. (2004) [67], and lauric acid from *L. salivarius* has the ability to remove biofilm, as suggested by Stenz et al. (2008) [68]. Monoterpenes, such as 1,8-cineole, are characterized by a broad spectrum of activity [11]. However, it is crucial to link these functions to the research technique used. Functional analysis must be supported by appropriate validation, to confirm that the observed antifungal activity comes from organic acids, it is necessary to neutralize the pH of the postbiotic. This aspect was demonstrated for postbiotics derived from *Lactobacillus* spp. RM1, where this treatment reduced activity by 50% [38]. Furthermore, the choice of sample preparation method, e.g., freeze-drying, which eliminates hydrogen peroxide [60], significantly narrows the interpretation of antimicrobial activity to non-volatile and thermally stable compounds, which is a key limitation when comparing results. The development of postbiotics, therefore, depends on the standardization of complex procedures, which will ensure the reproducibility of results and enable reliable functional validation of these promising biopreparations.

#### 4.1.3. Other Analytical Methods

Analysis of FTIR spectra showed that shifts in the hydroxyl (~3200–3400 cm^−1^) and carboxyl (~1600 cm^−1^) regions indicate the formation of intermolecular hydrogen bonds and electrostatic interactions between the bioactive components of postbiotics and the alginate polymer matrix, which directly contribute to the cohesion and stability of the coating [11]. Precise characterization of the chemical composition provides the basis for the next stage of research, which is to verify the biological efficacy of these preparations in in vitro and in situ systems [60].

### 4.2. Postbiotic Effectiveness Analysis

Assessing and verifying the functional potential of postbiotics requires a multi-stage efficacy evaluation: from preliminary in vitro tests to validation in target food matrices. This evaluation includes a wide range of methods that allow for the analysis of antimicrobial, antioxidant, and other functional properties [60].

#### 4.2.1. Antimicrobial Activity

Primary methods for assessing antibacterial efficacy include agar diffusion methods, such as the disc diffusion assay (DDA) and its variant, the well diffusion assay (WDA), which have been used in numerous studies [11,14,33,36,43,60,69].

Evaluating the potential applications of postbiotics in the food industry requires testing their activity against several key pathogens and microorganisms that cause food spoilage. Studies typically cover key foodborne pathogens that pose a serious threat to public health, such as *L. monocytogenes* [11,33,36,60,69], *E. coli* [11,33,43,69,70], *S. aureus* [11,33,43,69], *C. botulinum* [14], *Salmonella* spp. [11,33,43], *Pseudomonas aeruginosa* [43,70], *Bacillus cereus* [11,43], and *Proteus mirabilis* [70]. Following an initial assessment using diffusion methods, the next step is to apply methods that allow for a more precise determination of inhibitory concentrations.

After preliminary screening using diffusion methods, which allow for qualitative assessment, broth dilution methods are used to obtain precise quantitative data. These methods are crucial for the precise determination of the minimum inhibitory concentration (MIC) and minimum bactericidal concentration (MBC), which is the most accurate method for assessing the antimicrobial activity of postbiotics [33,36,43].

Davarzani et al. (2024) [33] evaluated the activity of postbiotics derived from *L. acidophilus* (LA) and *L. plantarum* (LP) strains and their freeze-dried mixture. Postbiotics from LA showed a stronger effect than mixture fractions. *L. monocytogenes* was the most sensitive to growth inhibition at 4.6 μg/mL LA, while *S. aureus*, *E. coli*, and *S.* Typhimurium required 6.25, 6.50, and 7.0 μg/mL, respectively. The combination of fractions significantly increased antibacterial activity (*p* < 0.05), reducing MIC/MBC to ≈3.75 μg/mL for *L. monocytogenes*, 4.25 μg/mL for *S. aureus*, and 5.0 and 6.75 μg/mL for *E. coli* and *S.* Typhimurium.

While MIC and MBC values provide fundamental data on the intrinsic activity of an antimicrobial substance, their direct translation to food conditions is limited. Therefore, the concept of minimum effective concentration (MEC) is introduced in application studies. This is a measure used to assess activity in complex food matrices such as meat or milk. MEC provides more practical information than MIC because it considers potential interactions with food components that may weaken or enhance the effect of bioconservatives. Moradi et al. (2019) [60] demonstrated that the MEC of postbiotics from *Lactobacillus* spp. depends on the matrix: for *L. salivarius*, the MEC was 5 mg/mL in broth, 15 mg/mL in milk, and 30 mg/mL in minced beef; for *L. acidophilus* LA5, the MEC was 15 mg/mL in broth and 45 mg/mL in both milk and beef. The analysis demonstrated that the efficacy of postbiotics strongly depends on the matrix: higher concentrations were required in food models because food components—particularly proteins and fats—can bind or sequester antimicrobial compounds, reducing their bioavailability and activity. In practice, this means that MIC/MBC results obtained in vitro must be verified under actual industrial conditions [60].

#### 4.2.2. Antioxidant Activity

The antioxidant potential of postbiotics is measured using tests based on free radical scavenging potential [33]. One of the most commonly used tests is the DPPH (2,2-diphenyl-1-picrylhydrazyl) radical test. The ABTS (2,2′-azino-bis(3-ethylbenzothiazoline-6-sulfonic acid)) cationic radical test is also widely used [11,33,43,71,72]. These analytical techniques provide a quantitative measure of antioxidant potential, which is ultimately derived from specific biochemical compounds within the postbiotic preparations. The antioxidant activity of postbiotics from LAB is often attributed to their being a source of antioxidant compounds with radical scavenging potential [72]. This activity is directly correlated with the presence of phenolic and flavonoid compounds [71].

In addition to the well-known role of phenolic compounds, postbiotics contain many other bioactive compounds that contribute to their antioxidant properties. The well-known antioxidant butylated hydroxytoluene (BHT) has been identified in postbiotics from *B. bifidum* [11]. Neutral exopolysaccharides isolated from *L. plantarum* have shown excellent radical scavenging ability [73]. In addition, *S. cerevisiae* can biosynthesise the natural antioxidant ergothioneine [74].

The DPPH test is used as a key reference point for quantitatively determining and comparing the free radical scavenging activity of postbiotics. The characteristics associated with this designation are essential for the selection of postbiotics for use in functional foods. The postbiotic derived from *L. acidophilus* showed a significantly higher percentage of DPPH removal (58.53%) compared to the postbiotic derived from *L. plantarum* (47.35%) [33]. Postbiotics derived from *Bifidobacterium* spp. showed DPPH radical scavenging activity ranging from 50.28 to 51.56 mg TEAC/100 mL [11]. In contrast, in this study, the combination of postbiotics from *Bifidobacterium bifidum* DSM 20,456 and BB12 showed the highest activity in its group, amounting to 51.56 ± 1.63 mg TEAC/100 mL [11].

#### 4.2.3. Other Functional Properties

The ability of postbiotics to remove bacterial biofilms, including those formed by *L. monocytogenes*, is quantitatively assessed using the crystal violet test [60]. This method allows for the determination of the total biofilm biomass and the assessment of the effect of the tested preparation on the adhesion and structural stability of the biofilm, but it does not allow for the differentiation between changes in the number of living cells and changes in the amount of extracellular matrix; therefore, its interpretation should be supplemented with other tests [75]. In order to assess the safety of potential applications of postbiotics, their cytotoxicity towards eukaryotic cells, e.g., L929 fibroblasts, is tested using a test using a 3-(4,5-dimethylthiazol-2-yl)-2,5-diphenyltetrazolium bromide (MTT) assay, which measures cell metabolic activity [60,76]. This test is commonly used as an indicator of cell viability and as a complementary tool for assessing antimicrobial activity [77]. In order to distinguish the mechanisms of action of cell-free supernatant (CFS) components, pH neutralization is used, whereby loss of activity after pH equalization indicates the dominant role of organic acids, while its maintenance suggests the involvement of pH-independent compounds such as peptides or bacteriocins [78]. Further verification of the nature of these compounds is provided by enzymatic treatment (e.g., with proteinase K). The disappearance of activity after the enzymatic process is strong evidence of the protein/peptide nature of the active substance, which has direct technological consequences (stability in food matrices, sensitivity to gastrointestinal enzymes, the need for encapsulation or formula modification [79].

## 5. The Use of Postbiotics in Various Food Matrices

The use of different strains for the production of postbiotics affects their activity in different food matrices: preparations derived from *Pediococcus* (especially *P. acidilactici*) showed the strongest effects in meat models, especially when used in combination with additional agents (e.g., chitosan) or applied at high concentrations, achieving multi-log reductions in *L. monocytogenes* and *S.* Typhimurium in frankfurters and chicken fillets [30,31]. Preparations from the *Lactobacillaceae* family showed a broad spectrum of activity and usefulness in coatings, marinades, and dairy products, but their effectiveness was more variable and in many cases required higher concentrations (e.g., 20–40%) or specific forms of application to yield significant reductions in real food matrices [12,34,36,40,80]. Postbiotics produced from yeast (*S. cerevisiae* var. *boulardii*) generally showed only small inhibition zones in diffusion tests on meat, suggesting lower antibacterial efficacy compared to selected postbiotics in these applications [43].

In general, minimum effective concentrations (MEC) are considerably higher in complex matrices such as milk or minced meat than in in vitro media/incubations. This matrix effect, driven by binding to proteins and fats and by diffusion barriers, reduces the availability and activity of bioactive compounds, which explains the need for higher doses in food [80,81]. Among the strains analyzed, the most consistent and strong effects were shown by postbiotics derived from *L. plantarum*, *L. rhamnosus*, and *P. acidilactici*, whereas preparations based on *Lactococcus lactis* and *L. casei* displayed more variable activity that depended strongly on matrix and concentration.

**Figure 2:**
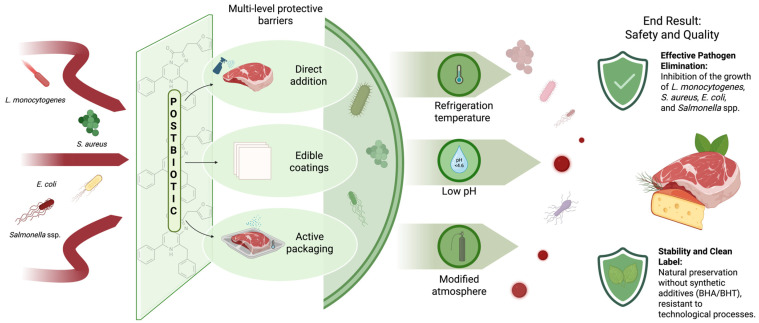
Postbiotics and hurdle technologies—a conceptual model for increasing food safety. Created in BioRender. Zakrzewski, A. (2026) https://BioRender.com/741d7j8 (accessed on 21 January 2026).

The most promising application technologies are surface coatings, active packaging systems, and combined formulas (e.g., postbiotic + chitosan) (Figure 2). These approaches increase local concentrations of the antimicrobial agent and can exploit synergistic mechanisms of action; however, because effectiveness varies significantly between matrices, pilot studies are required for each specific product to determine MEC, perform sensory evaluation, and assess shelf life [30,31,42,81].

Below, we summarize representative findings in two major food categories—meat and dairy—that illustrate the strain- and matrix-dependent behaviour described above.

### 5.1. Meat and Meat Products

Meat and meat products are a valuable source of complete protein, vitamins, and minerals, but due to their high water and nutrient content, they are highly susceptible to oxidation and microbial contamination, which leads to reduced quality and a shorter shelf life [4]. In order to limit the degradation of raw materials and extend the shelf life of meat products, chemical additives such as nitrates, nitrites, and synthetic antioxidants have been used for many years. Although they effectively inhibit oxidation processes and the growth of undesirable microbiota, their use is increasingly restricted due to potential health risks and growing consumer aversion to chemical preservatives [3]. One of the alternative and increasingly researched solutions in the meat industry is the use of postbiotics [82,83]. Their addition to meat products can be an effective strategy for limiting adverse oxidative changes in lipids and proteins, and consequently contribute to improving the shelf life, safety, and health benefits of meat products [84].

Several studies have evaluated the antimicrobial properties of postbiotics in meat matrices such as mutton [85], beef [12,40,60,80,81], chicken breast fillets [30], frankfurters [31], emulsion-type sausage [32], and hot dogs [86] (Table 2). Moradi et al. (2019) [60] evaluated the potential of postbiotics derived from LAB (*L. acidophilus* LA-5, *L. casei* 431, and *L. salivarius*) to inhibit the growth of *L. monocytogenes* both in vitro and in ground beef. The authors demonstrated that the extracellular supernatants of all three strains exhibited significant antimicrobial activity, stable over a wide pH range, and were partially resistant to temperature. Postbiotics obtained from *L. salivarius* showed the highest efficacy in inhibiting the growth of *L. monocytogenes* in meat, achieving a growth inhibition zone of nearly 25 mm, which indicates their potential for use as natural, safe biopreservatives in meat products. Studies on beef samples showed that a marinade containing tenfold concentrated CFS of *L. plantarum* inhibited the growth of *S.* Typhimurium, *L. monocytogenes*, *S. aureus* ATGC 29213, and *E. coli* in beef samples marinated under refrigerated conditions for 14 h, as confirmed by an inhibition zone of 24.89 ± 2.82 mm; 20.39 ± 3.57 mm; 20.53 ± 2.32 mm [80]. In contrast, Hartmann et al. (2011) [81] reported that CFS produced by various LAB strains (*Enterococcus sp., L. curvatus, L. plantarum, L. sakei, L. lactis, Leuconostoc carnosum, P. acidilactici, Staphylococcus sciuri*) used in concentrations ranging from 2% to over 10% reduced the population of *L. monocytogenes* by 2.30 log CFU/g in minced beef stored for 6 days at 4 °C. Beristain-Bauza et al. (2017) [40] observed that the addition of CFS *L. sakei* to whey protein coatings reduced the number of *L. monocytogenes* by 1.40 log CFU/g on fresh beef after 5 days of storage in a refrigerator. The antimicrobial properties of postbiotics were also confirmed by Valipour et al. (2024) [12], who observed significant anti-listerial activity of a postbiotic obtained from *L. sakei* in beef tenderloin—the use of 40% postbiotic effectively inhibited the growth of *L. monocytogenes*, reducing its count by more than 3.00 log CFU/mL during 15 days of storage.

Another example is the use of postbiotics to extend the shelf life of chicken breast fillets [30] and other meat products [31,86]. Ünlü et al. 2016 [86] demonstrated that freeze-dried CFS powders containing bacteriocins (nisin, nisin-like compounds, curvacin A, curvacin L442, bavaricin MN) derived from various LAB strains (*L. lactis* subsp. *lactis* ATCC 11454, *L. lactis* subsp. *cremoris* ATCC 14365, *L. lactis* BFE 920, *L. bavaricus* MN, *L. curvatus* LTH 1174, *L. curvatus* L 442) and applied to the surface of sausages, led to a significant reduction (4.00 log CFU/mL compared to the control sample) of *L. monocytogenes* during 4 weeks of storage at 4 °C, without any negative impact on the sensory properties of the product. The antibacterial effects of postbiotics have also been demonstrated in combination with chitosan [30,31]. In a study by İncili et al. 2021 [30], the use of postbiotics at concentrations of 10% and 50% obtained from *P. acidilactici* also reduced the number of *L. monocytogenes* by approximately 1.30 log CFU/g and *S*. Typhimurium by approximately 1.00 log CFU/g in meat samples. The same study also used a combination of postbiotics at an appropriate concentration with chitosan, which further enhanced the antimicrobial effect. The use of this system resulted in an additional reduction in the growth of *L. monocytogenes* by approximately 1.50 log CFU/g, regardless of the postbiotic concentration. Furthermore, the abundance of *S.* Typhimurium in samples containing 10% postbiotic with chitosan and 50% postbiotic with chitosan was significantly lower compared to control samples on day 0 and amounted to approximately 1.50 and 2.10 log CFU/g, respectively (*p* < 0.05) [30]. In another study, İncili et al. (2022) [31] analyzed the effect of chitosan enriched with the postbiotic *P. acidilactici* on foodborne pathogens. The results showed that the combination of postbiotic and chitosan (1.0% chitosan + 100% postbiotic) effectively inhibited the growth of *L. monocytogenes*, *S.* Typhimurium and *E. coli* on the surface of sausages, reducing their growth by an average of 5.00 log CFU/g, 4.00 log CFU/g, and 1.00 log CFU/g, respectively, on day 35 of storage compared to the control sample, while exhibiting a synergistic effect on the total viable count (TVC) of microorganisms, including LAB, mould and yeast, without affecting the pH and colour of the product. Similar effects in relation to *L. monocytogenes* were observed in studies by Bungenstock et al. (2021) [32], Junges Da Costa et al. (2021) [85], and Ünlü et al. (2016) [86]. The application of CFS *E. faecium* EO1 resulted in a reduction in *L. monocytogenes* (4.07 log CFU/g) in fresh sheep sausage after 30 days of storage at refrigeration temperature [85], while CFS obtained from *P. acidilactici* reduced the number of this pathogen already on day 0 [32]. Ünlü et al. (2016) [86] also reported a decrease in the number of *L. monocytogenes* after the application of freeze-dried, cell-free supernatants of LAB strains on the surface of frankfurters.

### 5.2. Dairy Products

Due to their popularity and widespread consumption, dairy products are an excellent matrix for enriching the diet with functional ingredients such as probiotics, prebiotics, plant fibres, and bioactive extracts [87]. In recent years, postbiotics have also been attracting increasing interest. They can be produced in culture media, food products, or the digestive tract, which makes dairy products a promising strategy for their application [39].

Previous studies have focused on evaluating the properties of postbiotics in dairy matrices such as yoghurt [33,35,37,39], cheese [36], and cheese whey [34] (Table 2). Sadighbathi et al. (2023) [39] aimed to evaluate the effect of postbiotics derived from *S. thermophilus* and *L. delbrueckii* subsp. *bulgaricus* on antioxidant activity, starter culture viability, and the quality of reduced-fat yoghurt during 22 days of storage. The authors highlighted the importance of using cheap and readily available raw materials for the production of postbiotics, including whey, which is a by-product of cheese production [39]. The results of the study showed that yoghurts enriched with postbiotics had increased antioxidant activity (18.71% inhibition on the 15th day of storage), which persisted throughout the storage period. In addition, sensory analysis showed that yoghurts containing postbiotics from *L. delbrueckii* subsp. *bulgaricus*, prepared in both whey and skimmed milk, received the highest acceptability ratings among panellists, confirming their potential as innovative functional products [39].

The results obtained are consistent with the observations of Yousefvand et al. (2024) [37], who used a freeze-dried postbiotic derived from *B. animalis* subsp. *lactis* BB12 to improve the antioxidant and physicochemical properties of reduced-fat yoghurt. The use of whey as a culture medium enabled the production of postbiotics with high biological activity, which translated into increased antioxidant activity of the products lasting for 21 days of storage at 4 °C and high sensory acceptability among consumers [37]. However, it is worth noting that yoghurt samples enriched with postbiotics showed a significant decrease in viscosity compared to the control sample, which is consistent with earlier studies by Pham et al. (2024) [35]. The authors suggest that this effect may result from the interaction of postbiotic components with milk proteins, leading to a weakening of the gel structure of the product. In contrast, Davarzani et al. (2024) [33] analyzed the antibacterial, antioxidant, and hypolipidemic properties of yoghurts enriched with postbiotics derived from *L. acidophilus* and *L. plantarum*. The postbiotics obtained showed significant antimicrobial activity, especially against Gram-positive bacteria such as *L. monocytogenes* (inhibition zone approx. 21.98 mm) and *S. aureus* (inhibition zone 20.83 mm). In the case of Gram-negative bacteria (*E. coli* and *S.* Typhimurium), significantly smaller growth inhibition zones were observed, with diameters of 18.94 and 15.26 mm, respectively [33].

Similar observations were also reported by Shahverdi et al. (2023) [69] and Hadadfar et al. (2025) [36], who reported that postbiotics, especially those derived from *L. plantarum*, exhibit particularly strong antimicrobial activity against Gram-positive bacteria such as *L. monocytogenes* and *S. aureus* [36]. In contrast, the activity against Gram-negative bacteria, such as *E. coli*, was moderate and comparable between the strains studied [88]. Davarzani et al. (2024) [33] showed in a rat model that consumption of yoghurt enriched with postbiotics derived from *L. acidophilus* and *L. plantarum* significantly reduced serum LDL cholesterol levels (*p* < 0.05), suggesting the potential health benefits of these products.

Another dairy product in which postbiotics are used is high moisture mozzarella cheese (HMMC), in which postbiotics obtained from *B. animalis* BB12 and *L. acidophilus* LA5 strains were tested for use as natural preservatives [34]. The authors used whey containing postbiotics derived from the above-mentioned microorganisms as a preservative, which extended the shelf life of the cheese. Both individual postbiotic solutions and their combinations exhibited antimicrobial activity, with some individual postbiotics showing stronger activity than their mixtures. The shelf life of HMMC was extended to 8 days, which is mainly attributed to the ability of the postbiotics used to inhibit the growth of mesophilic and psychrophilic bacteria. The postbiotic *L. acidophilus* LA5 (P-LA5) showed the strongest effect against mesophilic bacteria, reducing their growth by approximately 1.00 log CFU/g, while *B. animalis* BB12 effectively inhibited the growth of psychrophiles by 1.50 log CFU/g, maintaining the effect until the 16th day of storage [34].

### 5.3. Other Applications in Food Matrices and Bioactive Packaging

Postbiotics can be used not only as antimicrobial agents in meat, meat products, and dairy products, but also as natural ingredients in a variety of food matrices. In this context, scientific research to date has focused mainly on their antifungal properties and their use in bioactive films and composite yeast films (Table 2) [38,40,41,42,43,70,89,90,91].

Liu et al. (2023) [41] conducted research on the antifungal properties of CFS obtained from the *L. pentosus* 86 strain. It was shown that CFS from this strain effectively inhibited the growth of *Alternaria gaisen* (antimicrobial activity from 85.9% to 89.9%), while maintaining its stability after heat treatment and exposure to proteases. At the same time, it was observed that the antifungal activity of CFS weakened with longer incubation times, which is confirmed by earlier scientific reports [70,89]. This phenomenon can be explained by the relationship between the production and consumption of bioactive metabolites during the growth of microorganisms, which indicates a close relationship between antimicrobial activity and the phase of their development [38].

Mohammadi et al. (2022) [42] prepared bioactive packaging films based on bacterial nanocellulose (BNC) enriched with postbiotics obtained from a commercial bacterial preparation. The postbiotics contained various chemical compounds, such as fatty acids, aldehydes, alkanes, hydrocarbons, fatty acid esters, and propionic acid. They were responsible for effective antibacterial activity against pathogens such as *L. monocytogenes*. It was shown that the addition of postbiotics to BNC film effectively reduced the growth of microorganisms (inhibition zone of approx. 20 mm on average), slowed down oxidation processes, and extended the shelf life of food products. Previous studies by Rouxel et al. (2020) [90] and Beristain-Bauza et al. (2016) [91] also confirmed the antibacterial activity of postbiotics produced from *L. rhamnosus* against various bacterial pathogens in vitro and in whey protein coatings, which is attributed to the presence of lactic acid and bacteriocin-like compounds. These findings suggest that postbiotic-based active packaging should be regarded as a tailored technology: selection of the producing microorganism and optimization of the polymeric matrix must be made with respect to the specific microbial risks and quality targets of the intended food application.

In contrast, Abbasi et al. (2023) [43] demonstrated that composite yeast films containing bacterial cellulose, carboxymethylcellulose, and glycerol are highly soluble in water and have favourable preservative properties. The edible coating developed by the authors, based on *Malva sylvestris* mucilage and metabolites derived from *S. cerevisiae* var. *boulardii* ATCC MYA-796, has been shown to extend the shelf life of lamb by limiting chemical and microbiological changes during storage. For Gram-positive bacteria (*B. cereus, Listeria innocua, S. aureus)*, growth inhibition zones of 20.28 mm were obtained in the disc diffusion agar (DDA) test and 35.94 mm in the well diffusion agar (WDA) test, while for Gram-negative bacteria (*Salmonella* Typhi, *E. coli*, *P. aeruginosa*), these values were lower, at 14.80 mm and 17.85 mm, respectively [34]. Thus, whereas LAB-derived postbiotics tend to deliver pronounced antibacterial effects, yeast-derived metabolites in composite coatings appear to act more through improving oxidative stability and barrier properties—complementary mechanisms that together extend product shelf life.

Similar conclusions were reached by Beristain-Bauza et al. (2017) [40], who evaluated the antimicrobial activity of whey-protein films enriched with CFS derived from *L. sakei* used as packaging material for fresh beef. The developed films were shown to have an effective inhibitory effect against *L. monocytogenes* and *E. coli*. In an in vitro study, the addition of *L. sakei* CFS resulted in growth inhibition zones with a diameter of 4.83 mm for *E. coli* and 4.61 mm for *L. monocytogenes*. The effectiveness of the film was also confirmed in studies on a meat model stored under refrigerated conditions. The number of *E. coli* fell below the detection limit (<10 CFU/g) after 36 h, while the *L. monocytogenes* population was reduced by 1.4 log after 120 h of storage.

Collectively, postbiotic-enriched packaging can be effective across diverse carrier matrices, notably bacterial nanocellulose, whey-protein films, and edible yeast-based coatings, by combining antimicrobial action with antioxidative effects that together contribute to shelf-life extension. However, a closer comparison shows that, while antibacterial activity is a common outcome, the magnitude, spectrum, and persistence of inhibition depend strongly on both the microbial origin of the postbiotic and the physicochemical properties of the polymeric carrier.

## 6. Conclusions

From an industrial perspective, postbiotics have significant advantages over live probiotics and protective cultures: better stability, no live cells, and easier integration into existing processing and safety systems (including barrier technologies, edible coatings, and active packaging materials). At the same time, large-scale implementation is hampered by two main challenges: regulatory uncertainty due to the lack of uniform definitions and classifications, and the lack of standardized, recognized protocols for production, characterization, and potency testing. In order to translate the documented properties of postbiotics into safe, repeatable, and commercially viable applications in food, the priorities are standardization of definitions and quality requirements, development of validated methods for production and characterization (including analytical panels for key metabolites), systematic validation of efficacy in real matrices, and comprehensive toxicological and technological safety assessment.

An additional critical barrier to industrial implementation is production costs. Large-scale production of postbiotics—and the final processing steps, such as freeze-drying—account for a significant portion of the total process costs. In response, spray drying is increasingly being considered as a potentially more economical alternative to freeze-drying; however, successful implementation requires advanced process parameter optimization, which entails further investment. In addition to costs, there are important technological challenges to be addressed: interactions between postbiotic compounds and food matrix components, and the impact of further processing operations on the stability and activity of postbiotics. Furthermore, the addition of postbiotics may affect the organoleptic properties of the product, as some metabolite fractions or carriers used in encapsulation may modify the taste, aroma, or overall consumer perception. Therefore, development work should include not only cost reduction strategies and scalable drying strategies (with thorough process optimization), but also formulation and encapsulation approaches that preserve activity while minimizing the impact on sensory properties, as well as dedicated sensory and shelf-life testing in target products.

Postbiotics are a promising strategy for reconciling expectations for clean food labelling with the need to maintain high levels of microbiological safety. Available data show that postbiotics produced mainly by lactic acid bacteria exhibit a broad spectrum of antibacterial activity against key foodborne pathogens such as *L. monocytogenes*, *S. aureus*, *E. coli*, and *Salmonella* spp. In addition, postbiotics have antioxidant properties, which in laboratory studies and in complex food matrices translate into a significant reduction in the number of microorganisms and a delay in spoilage processes. In meat and meat products, a reduction in several logarithms and improved oxidative stability have been observed without any negative impact on sensory quality. In dairy products, postbiotics increase microbiological stability and enhance antioxidant activity, and in some cases, potential health benefits have also been reported.

The effectiveness of postbiotics depends largely on the type of food matrix. The activity observed in model systems such as broths does not translate directly to food products, as matrix parameters (including fat and protein content, pH, and water activity) significantly modify the minimum effective concentrations and dynamics of action. Therefore, the design of applications requires validation in specific products and adaptation of doses and formulations to the conditions of real food systems.

The chemical profile and biological activity of postbiotics are determined by production and processing parameters: strain selection, fermentation substrate, inactivation method, and preservation and dosing methods. The wide variety of metabolites detected by chromatographic and spectroscopic techniques explains the variability in antibacterial and antioxidant activity between studies and highlights the need for strict control of production processes and full reporting of technological parameters.

Therefore, research, development, and regulatory initiatives must go beyond basic efficacy studies to include cost analyses, scalable final processing (including optimized drying), formulation/encapsulation development, sensory impact assessment, and robust safety testing in the context of intended food applications.

## Figures and Tables

**Table 1:** Microbial cultivation conditions used in postbiotic production and methods for their production and application.

Species	Culture Medium	Temperature [°C]	Incubation Time [h]	Supplement	Culture Conditions	Postbiotic Production (1)/Application (2)	Source
*S. thermophilus*	Skimmed milk	40	68	-	Aerobic	Freeze-drying (−60 °C, 0.0046 mbar, 48 h) Directly use	[39]
	Whey	39.6	68				
*L. delbrueckii* subsp. *bulgaricus*	Skimmed milk	46	64				
	Whey	42.1	68				
*B. animalis* subsp. *lactis*	Skimmed milk	30	48	Lactose (0.18%)		Freeze-drying (−60 °C, 0.0650 mbar, 48 h) Directly use	[2,37]
	Whey	30	40.8	Lactose (1.5%)			
*L. acidophilus*	Whey	42	35.5	Lactose (3.9%)			[35]
	Skimmed milk	30	48	Lactose (1.0%)			
*L. acidophilus* BLAC 258	Skimmed milk	37	48	Yeast extract (1.0%)		Freeze-drying (NA) Dissolve 1 g in 1 mL of phosphate-buffered solution	[33]
*L. plantarum* BLP 272							
*L. plantarum*	MRS broth	37	24	-	Anaerobic	Freeze-drying (−83 °C, 0.0026 mbar) CFS	[36]
*L. casei*							
*L. acidophilus*	Whey	37	36	Yeast extract (1.0%)	Aerobic	Postbiotic solution Directly use	[34]
*B. animalis* BB12							
*P. acidiactici*	TSB	37	24	-		NA Diluted with sterilized distilled water to concentrations of 10% and 50%	[30]
*P. acidilactici*	MRS broth	37	48		Anaerobic	Postbiotic solution Preparation of 100% and 50% postbiotic concentrations	[31]
*L. sakei*	MRS broth	37	20			Freeze-drying (NA) Rehydration in distilled water	[40]
*L. pentosus*	MRS broth	37	NA		Aerobic	Postbiotic solution Direct addition to the microbiological substrate	[41]
*Lactobacillus* ssp.	MRS broth	37	24			Freeze-drying (15–18 h) Postbiotic solution	[38]
*S. cerevisiae* var. *boulardii*	YMB	37	24–48			NA	[43]
*Pediococcus* ssp.	MRS broth	30	48		Anaerobic	Postbiotic solution Dissolution in phosphate buffer	[32]
*Lactococcus* ssp.							
*P. acidilactici*							
*P. pentosaceus*							
*L. lactis*							
Commercially available strain preparation (NA)	MRS broth	37 ± 1	24		Aerobic	Freeze-drying (−40 °C, pump pressure: 100 mTorr) Dissolution in phosphate buffer with saline solution	[42]

Abbreviations: MRS broth—De Man, Rogosa, and Sharp broth; TSB—Tryptic Soy Broth; YMB—Yeast Mannitol Broth; NA—no data available; CFS—cell free supernatant; Taxonomic nomenclature follows ICNP; the abbreviation “subsp.” is used consistently in the manuscript. Original forms from cited sources have been retained where applicable.

**Table 2:** Application of postbiotics in food matrices (meat, meat products, and dairy products) and in vitro models.

No.	Matrix/Model	Strain	Method of Application	Effect				Source
				Preparation Concentration	Duration/Conditions	Result [log CFU]/Effect	Comments	
1.	Chicken breast fillets	*P. acidilactici*	Liquid (coating)	Postbiotic 50%	15 days	*S.* Typhimurium: 4.33 → 3.54 log CFU/g (reduction ≈ 0.79 log) *L. monocytogenes*: 7.20 → 6.31 log CFU/g (reduction ≈ 0.89 log)	No effect on pH or colour	[30]
				Postbiotic 50% + chitosan 1%		*S.* Typhimurium: 4.33 → 2.96 log CFU/g (reduction ≈ 1.37 log) *L. monocytogenes*: 7.20 → 4.12 log CFU/g (reduction ≈ 3.08 log)	Synergy of chitosan + postbiotics	
2.	Frankfurters	*P. acidilactici*	Solution	Chitosan 1.0% + postbiotic 100%	35 days	Reductions relative to controls: *L. monocytogenes* ≈ 5 log, *S. typhimurium* ≈ 4 log, *E. coli* ≈ 1 log	Sustained effect during storage. No effect on pH and colour	[31]
3.	Beef fillet	*L. sakei*	Aerosol	Postbiotic 20%	15 days	No significant decrease compared to the control sample	Aerosol application may support antibacterial packaging concepts	[12]
				Postbiotics 40%		*L. monocytogenes:* 6.20 → 3.30 log CFU/g (reduction ≈ 2.90 log)		
4.	Mutton sausage	*E. faecium*	Suspension added to sausage stuffing	Postbiotic 5%	30 days	*L. monocytogenes:* 36 isolates inhibited growth, forming inhibition zones ranging from 1.2 to 6.2 mm.	-	[85]
				Postbiotics 10%				
5.	Fresh beef	*L. sakei* *L. plantarum* *L. rhamnosus*	Suspension	Addition of marinade to postbiotic in a ratio of 5:1	14 h	Postbiotic from *L. plantarum:* inhibition zones →*S.* Typhimurium (24.89 mm), *Pseudomonas* spp. (19.5 mm), *S. aureus* (18.93 mm) and *Shigella sonnei* (15.63 mm). Postbiotic from *L. sakei*: inhibition zones → *S. aureus* (22.77 mm), *E. coli* (20.83 mm), *L. monocytogenes* (22.90 mm) Postbiotic from *L. rhamnosus*: inhibition zones → *E. coli* (17.15 mm), *S. typhimurium* (18.89 mm), *S. sonnei* (14.54 mm), *P. fluorescens* (15.59 mm), *L. monocytogenes* (19.51 mm), *S. aureus* (21.83 mm)	Marinade with added CFS may have potential use as a biopreservative for fresh beef.	[80]
6.	Ground beef, Whole Milk, BHI	*Enterococcus* sp., *L. curvatus*, *L. plantarum*, *L. sakei*, *L. lactis*, *L. carnosum*, *P. acidilactici*, *S. sciuri*	Solution	0.1, 0.2, 0.4, 0.6, 0.8, 1.0, 1.2, 1.4, 1.6, 1.8, 2.0, 4.0, 6.0, 8.0, and 10.0% of MEC		*L. monocytogenes:* MEC of the fermentates is higher in milk and ground beef; however, their effectiveness varied significantly depending on the matrix	Transitional anti-listerial activity of all preparations. Matrix-dependent variability of MEC values.	[81]
7.	Hot dogs	*Lactococcus lactis* subsp. *lactis*, *Lactococcus lactis* subsp. *cremoris*, *L. lactis*, *Lactobacillus bavaricus*, *L. curvatus*	Solution/powder	0.6 g/bag	1 month	Initial concentration of *L. monocytogenes* in samples ≈ 10^9^ CFU/mL Bacteriocin from *L. curvatus* → No differences Bacteriocin from *L. lactis* subsp. *cremoris* → ≈ 2 log CFU/mL, Bacteriocin from *L. lactis* supsp. *lactis* and *L. lactis* → ≈ 3 log CFU/mL	-	[86]
8.	Fresh beef	*L. sakei*	Coating	Postbiotic (unspecified concentration)	1.5–5 days	*E. coli:* <10 CFU/g after 36 h. *L. monocytogenes:* reduction—1.4 log CFU/mL after 120 h.	Increased elimination of *E. coli* in the meat model	[40]
9.	Lamb meat	*S. cerevisiae* var. *boulardii* ATCC MYA 796 + Polysaccharide mucilage from forest mallow seeds (*Malva sylvestris*)		Postbiotics (2%, 4%, 6%, 8%, 10%)	Diffusion tests	Inhibition zones: *E. coli*—4.83 mm. *L. monocytogenes*—4.61 mm (in one test). DDA/WDA: Gram+—20.28 mm/35.94 mm; Gram−—14.80 mm/17.85 mm.	The results indicate a stronger effect against Gram+ bacteria.	[43]
10.	Emulsion type sausage	*Pediococcus* spp., *Lactococcus* spp., *Pediococcus acidilactici* LMQS 154.1, *L. pentosaceus* LMQS 331.3 *L. lactis* DSM 20729		Postbiotics	NA	No significant growth inhibition was observed Concentrated bacteriocin preparations have shown potential in inhibiting *L. monocytogenes*	Bacteriocin concentrate showed anti-listerial potential	[32]
11.	Ground meat, Pasteurized milk	*L. acidophilus*, *L. casei*, *L. salivarius*	Solution	Postbiotic	6 days	Zones inhibiting the growth of *L. monocytogenes:* *Lactobacillus acidophilus* → (26 mm) *L. casei* → (18 mm) *L. salivarius* → (22 mm)	Potential for biofilm removal. Spectrum of activity at different pH values.	[60]
12.	Yoghurt	*S. thermophilus*, *L. delbrueckii* subsp. *bulgaricus*	Powder	Postbiotics	Storage period (sensory evaluation)	High antioxidant activity (≈18.71%); no significant effect on the overall properties of yoghurt	Favourable physical and chemical properties (appropriate level of syneresis, water retention); high sensory acceptability	[39]
13.		*B. animalis* subsp. *lactis* BB12		Postbiotics	During storage; assessment up to 10 days	Antioxidant activity maintained over time (6.30 → 5.59 → 4.59 → 2.67%, respectively, in skimmed milk, skimmed milk and whey, whey and control)	High consumer acceptance after 10 days	[37]
14.		*L. acidophilus LA5*			Up to 21 days (assessment after 21 days)	Increased dry matter and ash content (0.75 g/100 g); antioxidant activity ≈ 5.94%	Reduction in syneresis (≈20.65% in the whey sample); after 21 days, higher acidity and faster growth of *L. bulgaricus*	[35]
15.		*L. acidophilus*, *L. plantarum*			In vitro tests/in vivo studies (rats)	Strong antibacterial activity against *S. aureus*, *E. coli*, and *S. typhimurium.* DPPH: *L. acidophilus* 58.53% vs. *L. plantarum* 47.35% (*p* < 0.05) reduction in cholesterol in rats (91.75 → 68.81)	Both postbiotics resulted in lower DPPH values than in the control sample (75.63%); in vivo hypolipidemic effect.	[33]
16.	Cheese	*L. plantarum*, *L. casei*		Postbiotic (concentration 5%, 20%)	Antibiotic tests (in vitro)	Postbiotic *L. plantarum* (20%) against *L. monocytogenes*—inhibition zone 30.67 mm. *L. casei* (5%) weakest inhibition vs. *S. aureus*—8.63 mm	Differences in effect depending on the strain and concentration of the postbiotic	[36]
17.	In vitro	*Lactobacillus* spp.	-	-	Antibiotic tests (in vitro)	Complete inhibition of aflatoxin B1 and ochratoxin production; reduction in mycelium weight from 37.4 to 8.2 (g/250 mL)	Strong antimicrobial activity of CFS (examples: activity against *B. subtilis*, *Salmonella* sp.)	[38]
18.	High-Moisture Mozzarella	*L. acidophilus* LA5 *B. animalis* BB12	Suspension	-	Storage—up to 16 days; shelf-life assessment	Extension of cheese shelf life to 8 days; reduction in the growth of mesophiles (≈1 log CFU/g) and psychrophiles (≈1.5 log CFU/g)	LA5 strongest against mesophiles; BB12 effective against psychrophiles; effect maintained until day 16	[34]
19.	Packaging material based on bacterial nanocellulose	Commercially available strain preparation	Solution	-	Coating application/in vitro tests	Significant antimicrobial activity against *S. aureus*, *L. monocytogenes*, *S.* Typhimurium, *E. coli*, and moulds (*A. flavus*, *P. citrinum*)—inhibition zones ≈ 20 mm	Potential for use in active antibacterial packaging	[42]
20.	In vitro	*L. pentosus* 86	Coating	-	Antifungal tests (coating)	Strong antifungal activity of CFS/coating; activity 85.8–89.9% (antifungal inhibition)	Possible use in the protection of raw materials/products	[41]

Abbreviations: DDA—disc diffusion assay; WDA—well diffusion assay; MEC—minimal effective concentration of the cell-free supernatants for inhibition; NA—no data available.

## Data Availability

No new data were created or analyzed in this study. Data sharing is not applicable to this article.

## Abbreviations

The following abbreviations are used in this manuscript:

BLC	Bacteriocin-like Compounds
CFS	Cell-Free Supernatant
CFU	Colony-forming units
DDA	Disc Diffusion Assay
DPPH	2,2-diphenyl-1-picrylhydrazyl assay
EFSA	European Food Safety Authority
FTIR	Fourier Transform Infrared Spectroscopy
GC-MS	Gas Chromatography with Mass Spectrometry
HMMC	High Moisture Mozzarella Cheese
HPLC	High-Performance Liquid Chromatography
LAB	Lactic Acid Bacteria
MBC	Minimum bactericidal concentration
MEC	Minimum effective concentration
MIC	Minimum inhibitory concentration
MRS	De Man, Rogosa, and Sharpe Broth
NA	No Data Available
QPS	Qualified Presumption of Safety
TEAC	Trolox equivalent antioxidant capacity
TPC	Total Phenolic Content
TSB	Tryptic Soy Broth
TVC	Total Viable Count
WDA	Well Diffusion Assay
YMB	Yeast Mannitol Broth
